# High Initiation but Suboptimal Completion of Tuberculosis-Preventive Treatment Among People Living With HIV on Antiretroviral Therapy, Persistent Tuberculosis Incidence, and Programmatic Implications: Findings From a 2-Year Multicountry Cohort Study

**DOI:** 10.1093/ofid/ofag595

**Published:** 2026-10-13

**Authors:** L Chimoyi, S Ginindza, B A S Nonyane, C Mulder, A Bedru, N Kawaza, J E Golub, K Shearer, A Kennar, L Mvusi, K Vilakazi-Nhlapo, T Apollo, M Ncube, C Gwanzura, G Bisa, D Gadissa, T Mabuto, L Tsope, I Rabothata, N Ntombela, K Sithole, S Sisay, M Makgabo, R E Chaisson, G Churchyard, C J Hoffmann, V Chihota

**Affiliations:** Implementation Research Division, The Aurum Institute, Isando, South Africa; Implementation Research Division, The Aurum Institute, Isando, South Africa; Center for Tuberculosis Research, Johns Hopkins University, Baltimore, Maryland, USA; Department of TB Elimination and Health System Innovations, KNCV Tuberculosis Plus, The Hague, the Netherlands; KNCV Tuberculosis Plus, Addis Ababa, Ethiopia; TB/HIV Department, Clinton Health Access Initiative, Harare, Zimbabwe; Center for Tuberculosis Research, Johns Hopkins University, Baltimore, Maryland, USA; Center for Tuberculosis Research, Johns Hopkins University, Baltimore, Maryland, USA; Center for Tuberculosis Research, Johns Hopkins University, Baltimore, Maryland, USA; TB Cluster, National Department of Health, Pretoria, South Africa; TB Cluster, National Department of Health, Pretoria, South Africa; National TB and Leprosy Program, Ministry of Health and Child Care, Harare, Zimbabwe; National TB and Leprosy Program, Ministry of Health and Child Care, Harare, Zimbabwe; National TB and Leprosy Program, Ministry of Health and Child Care, Harare, Zimbabwe; Addis Ababa Health Bureau, Addis Ababa, Ethiopia; KNCV Tuberculosis Plus, Addis Ababa, Ethiopia; Implementation Research Division, The Aurum Institute, Isando, South Africa; Implementation Research Division, The Aurum Institute, Isando, South Africa; Implementation Research Division, The Aurum Institute, Isando, South Africa; Implementation Research Division, The Aurum Institute, Isando, South Africa; TB/HIV Department, Clinton Health Access Initiative, Harare, Zimbabwe; KNCV Tuberculosis Plus, Addis Ababa, Ethiopia; Implementation Research Division, The Aurum Institute, Isando, South Africa; Center for Tuberculosis Research, Johns Hopkins University, Baltimore, Maryland, USA; Implementation Research Division, The Aurum Institute, Isando, South Africa; School of Public Health, University of the Witwatersrand, Johannesburg, South Africa; Department of Medicine, Division of Infectious Diseases, Vanderbilt University, Nashville, Tennessee, USA; Implementation Research Division, The Aurum Institute, Isando, South Africa; Center for Tuberculosis Research, Johns Hopkins University, Baltimore, Maryland, USA; Implementation Research Division, The Aurum Institute, Isando, South Africa; School of Public Health, University of the Witwatersrand, Johannesburg, South Africa; Department of Medicine, Division of Infectious Diseases, Vanderbilt University, Nashville, Tennessee, USA

**Keywords:** cascade of care, digital tools, people living with HIV (PLWH), routine settings, tuberculosis preventive treatment

## Abstract

**Background:**

Tuberculosis-preventive treatment (TPT) is recommended for those at risk of tuberculosis, but despite encouraging trial data, evidence of its effectiveness from routine settings with diverse TPT regimens is limited. We describe routine TPT implementation outcomes in people living with human immunodeficiency virus (HIV; PLWH) to identify bottlenecks and opportunities for improvement along the cascade of care.

**Methods:**

From 2021–2023, we conducted a 24-month prospective cohort study of PLWH facilities in Ethiopia, South Africa and Zimbabwe. Sociodemographic and clinical data were collected at enrollment. During follow-up, we collected TPT initiation, TPT-related adverse effects, and completion data. A subgroup was allocated digital boxes as a proxy measure for assessing treatment completion. Tuberculosis diagnoses were confirmed from clinic records or symptom agnostic sputum testing using Xpert MTB/RIF assay and mycobacteria growth indicator tube (MGIT) culture at 12- and 24 months after enrollment.

**Results:**

We enrolled 2095 participants, mostly female (n = 1489 [71.1%]), with a median age of 42 years (interquartile range, 35–50 years). Approximately 60% (n = 1312) were considered eligible for TPT, and 1110 (84.6%) initiated TPT within 60 days of antiretroviral therapy initiation or refill during follow-up (rifapentine and isoniazid [3HP] in 625 and 6 or 12 months of isoniazid [6H/12H] in 487). TPT was completed in 55.4% (406 of 733). Mild symptoms compatible with TPT adverse effects were commonly reported in 3HP versus 6H/12H (190 [31.0%] vs 51 [11.0%], respectively; *P* < .001]. The tuberculosis incidence rate was 1.27/100 person-years (95% confidence interval, .95–1.69) during study follow-up in a mostly long-term antiretroviral therapy population and was lower for PLWH who completed TPT (adjusted incidence rate ratio, 0.39 [.20–.78]; *P* = .01).

**Conclusions:**

There were losses along the cascade, particularly at completion. In programmatic settings, we observed more potential mild symptoms from 3HP compared to 6H/12H use. Interventions to support TPT completion are needed.

## Teaser

Study showed that 3HP is well tolerated with high completion rates. Although programmatic tuberculosis-preventive treatment (TPT) completion was overestimated, adherence gaps persist, and incident TB occurred in 2.5%. TB programmes need to strengthen TPT prescribing, adherence support, monitoring, and integrated TB/HIV care.

The 2024 World Health Organization (WHO) global tuberculosis report estimated that approximately 23% of the global population had tuberculosis infection, with about 6% of these individuals progressing to tuberculosis disease. Among individuals with tuberculosis disease in 2023, an estimated 662 000 were people living with human immunodeficiency virus (HIV; PLWH), among whom an estimated 161 000 deaths occurred [1]. In 2023, according to the 2024 WHO global report, the overall tuberculosis incidence rates per 100 000 in Ethiopia, South Africa, and Zimbabwe were 146, 427, and 211, respectively [1]. The tuberculosis incidence rates among PLWH in these countries were 12, 230, and 127 per 100 000, respectively [1].

There is substantial evidence from studies conducted globally supporting the provision of tuberculosis-preventive treatment (TPT) with isoniazid to decrease progression to tuberculosis disease, reducing the risk of tuberculosis disease by 40% and the risk of death among PLWH [2, 3]. Based on these observations, TPT is a key tuberculosis control strategy recommended by the WHO [4]. Though recommended by the WHO since 1991, the delivery of TPT has generally been suboptimal. Reasons for low uptake include safety concerns, fears of selecting for drug resistance if tuberculosis disease is not fully excluded, and treatment duration [5]. While the WHO has reported progress in the delivery and uptake of TPT in PLWH [1], many settings continue to experience suboptimal provision and completion of TPT [5].

The WHO recommends TPT for all PLWH, provided that active tuberculosis disease has been ruled out using the WHO-recommended 4-symptom screen (any current cough, fever, night sweats, or weight loss), with further investigation where indicated [6]. National guidelines in Ethiopia, South Africa, and Zimbabwe recommend that all PLWH receive adherence counseling before TPT initiation, clinical monitoring for tuberculosis disease, and laboratory testing for liver toxicity at each antiretroviral therapy (ART) initiation and/or refill visit [7–9]. The guidelines also specify a duration of 2–6 months between ART refill visits in community-based differentiated service delivery models [7–9].

In recent years, shorter-course TPT regimens have been recommended, including 1-month and 3-month rifapentine and isoniazid combination regimens [10–14], with the 3 months of weekly rifapentine and isoniazid (3HP) recommended by the WHO as the preferred treatment. Successful scale-up in routine settings is dependent on identifying and addressing factors related to attrition along the TPT delivery cascade. We sought to describe routine TPT implementation and identify opportunities for improvement by evaluating TPT delivery among PLWH attending routine primary healthcare settings in Ethiopia, South Africa, and Zimbabwe.

## METHODS

### Study Design and Settings

The optimizing TPT delivery among PLWH (Opt4TPT) study was a multisite observational prospective cohort study of PLWH receiving routine HIV care in 9 primary healthcare facilities in Ethiopia, South Africa, and Zimbabwe. The selected facilities were primary care clinics with established tuberculosis programs and had ≥300 monthly HIV/ART clinic encounters and provided 3HP, 6 months of isoniazid (6H), and/or 12 months of isoniazid (12H) to their patients. The expected frequency of monthly ART initiation visits in all 9 healthcare facilities was between 39 and 74 visits. Similarly, 953–7103 refill visits were expected during the study enrollment period. National guidelines for each country stipulated that all PLWH without tuberculosis symptoms, tuberculosis, or liver disease or with a history of TPT history (>2–3 years) or tuberculosis treatment should receive TPT [7–9].

### Sample Size Considerations

The sample size estimates were based on the outcome of successfully completing 3HP or 6H/12H, and we used data from clinical trials indicating that completion could range from 65% to 97% [10, 15–18]. We hypothesized that we would observe 90% 3HP, and 65% 6H/12H completion rates in our setting. We aimed to enroll 2000 PLWH for the cohort study and to be able to monitor ≥1500 PLWH for completion, assuming that they were either initiating or refilling ART. We considered a conservative estimate of 15% loss to follow-up, which would enable us to estimate completion with a margin of ±3% for completion rates between 65% and 95% and a 2-sided 5% level of significance. This sample size was also sufficient to provide estimates of adverse effects within ±2% margin of error, if the rates of adverse effects ranged from 2% to 7%, as observed in prior studies [10, 15–18].

### Study Population

The Opt4TPT study was conducted among PLWH attending routine HIV care. TPT assessment, prescription, and monitoring were conducted by healthcare workers as part of routine care. From July to December 2021, healthcare workers referred potential participants attending routine HIV care visits to research staff in the selected healthcare facilities for screening and enrollment into the study. According to the study eligibility criteria, we enrolled adult participants (aged ≥18 years) newly initiated on ART or refilling ART in the study clinics regardless of their TPT history and followed them up for 24 months. Participants also had to be willing and able to participate in telephonic study visits, attend in-person clinic visits at 12 and 24 months after enrollment, and provide written informed consent.

### Study Procedures

Face-to-face study visits with study staff at months 3, 6, 12, and 24 were aligned with routine clinic visits for people being newly initiated on ART or returning for a refill. We also conducted telephonic visits at months 1, 2, and 21. At enrollment, we collected self-reported data on sociodemographic characteristics, as well as clinical and medical history. At baseline and during follow-up, we abstracted data on TPT initiation and completion and tuberculosis diagnoses from clinic files. TPT initiation and completion were defined as having a documented TPT start and stop date in the clinic files. Participants initiating TPT during the study implementation period were offered an evriMED1000 medication event reminder monitor (MERM) device to measure the opening of boxes as a proxy for monitoring uptake, with medication reminders switched off, until all devices were allocated.

At 12 and 24 months after enrollment, we collected sputum samples from all available participants for microbiological tuberculosis investigations. Positive Xpert MTB/RIF Ultra and mycobacteria growth indicator tube (MGIT) culture tests results confirmed tuberculosis diagnosis. In addition, information on tuberculosis symptoms (self-reported), deaths, and adverse effects/symptoms (reported to study staff and abstracted from clinic files) was collected for 3HP and 6H/12H. After study completion, we abstracted the total number of PLWH eligible for and prescribed TPT during the study period from country-specific electronic health records, such as district health information systems.

### Outcome Measures

The primary outcome was the proportion of participants who initiated TPT within 60 days of ART initiation or refill visit in routine health facilities. Since screening for TPT eligibility was conducted as part of routine care, for the purposes of this analysis we considered eligibility for TPT as having no documented evidence of prior TPT in the clinic records. A clinical record of TPT completion was defined as a record of dispensing a full course of the TPT regimen without a notation of premature discontinuation.

The secondary outcomes were (1) the proportion of PLWH completing a TPT dose by clinic record or digital pillbox, (2) the proportion missing TPT doses, and (3) the proportion on TPT who reported adverse effects or any symptoms. Exploratory outcomes were (1) the tuberculosis incidence rate during follow-up, (2) occurrences of asymptomatic tuberculosis 12 and 24 months after enrollment, and (3) the proportion of deaths among PLWH during the follow-up period. TPT completion as indicated by digital pillbox was defined according to the WHO guidelines of 80% of possible doses taken for 6H/12H and 90% of possible doses taken for 3HP, within an additional 33% duration extension to allow for completion [13]. Following these guidelines, treatment completion was defined as 146 doses within a maximum of 239 days for 6H, 292 doses within 476 days for 12H, and 11 doses within 120 days for 3HP. TPT adherence was measured using the electronic monitoring box. The TPT regimen was defined as the regimen recorded in the evriMED1000 database, and the first intake date was used as the TPT initiation date. One dose was counted for each calendar day that the device was opened for 6H/12H, while for 3HP a dose was counted as valid only if it was ≥4 days after the previous dose. The symptoms were reported either to study staff during a study visit or to healthcare staff during routine clinical care.

### Statistical Analysis

For continuous outcomes we summarized study cohort characteristics and outcomes using medians and interquartile ranges (IQRs). We described categorical variables using frequencies and proportions. Bar graphs were used to summarize the reported TPT-related symptoms and the TPT cascade. Cases with missing values were considered as baseline categories or nonevents and included in the denominators of categorical variables. We present primary, secondary, and exploratory outcome results as proportions. Poisson regression models were used to summarize and compare all-cause mortality rates between TPT regimens and evaluate predictors of TPT initiation, completion by MERM, and tuberculosis incidence, and results are presented as risk ratios with corresponding 95% confidence intervals (CIs).

Variables with a *P* value <.20 at the univariable level were included in the multivariable regression models but some variables considered important for their potential roles as confounders of selected outcomes (treatment initiation, completion, and incident tuberculosis cases) were included a priori regardless of the *P* value. The *P* < .20 threshold was used to avoid potentially excluding variables too early and to avoid overfitting. The tuberculosis incidence rate was calculated to assess the risk of tuberculosis over the follow-up period. Sensitivity analyses on TPT completion based on digital pillbox use were conducted to examine the robustness of parameter estimates in the case of missing data explained by challenges experienced using the digital pillbox. Statistical significance was determined at a 2-sided 5% threshold. We conducted all analyses using Stata 17 software [19].

### Patient/Public Involvement and Ethical Approvals

This study was designed without patient participation. It was approved by the University of the Witwatersrand Human Research Ethics Committee (no. 210212), the Institutional Review Board of Johns Hopkins University (IRB00280677), the Medical Research Council of Zimbabwe (MRCZ/A/2727), and the Addis Ababa Health Bureau Institutional Review Board (A/A/145/227). We also received approval from the local district research ethics committees in South Africa and permission from the healthcare facilities.

## RESULTS

During the enrollment period >9613 PLWH made ≥1 visits to a study clinic. Of these, 721 (7.5%) were initiating ART while 8892 (92.5%) were returning for/established on ART care (>6 months on ART). Of the total patient population in the study clinics, 2263 (23.5%; 754 in Ethiopia, 753 in South Africa, and 756 in Zimbabwe) were referred to study staff for study enrollment. Of those, 2095 (93%) were eligible for study participation and consented to participate (Figure 1).

**Figure 1.**
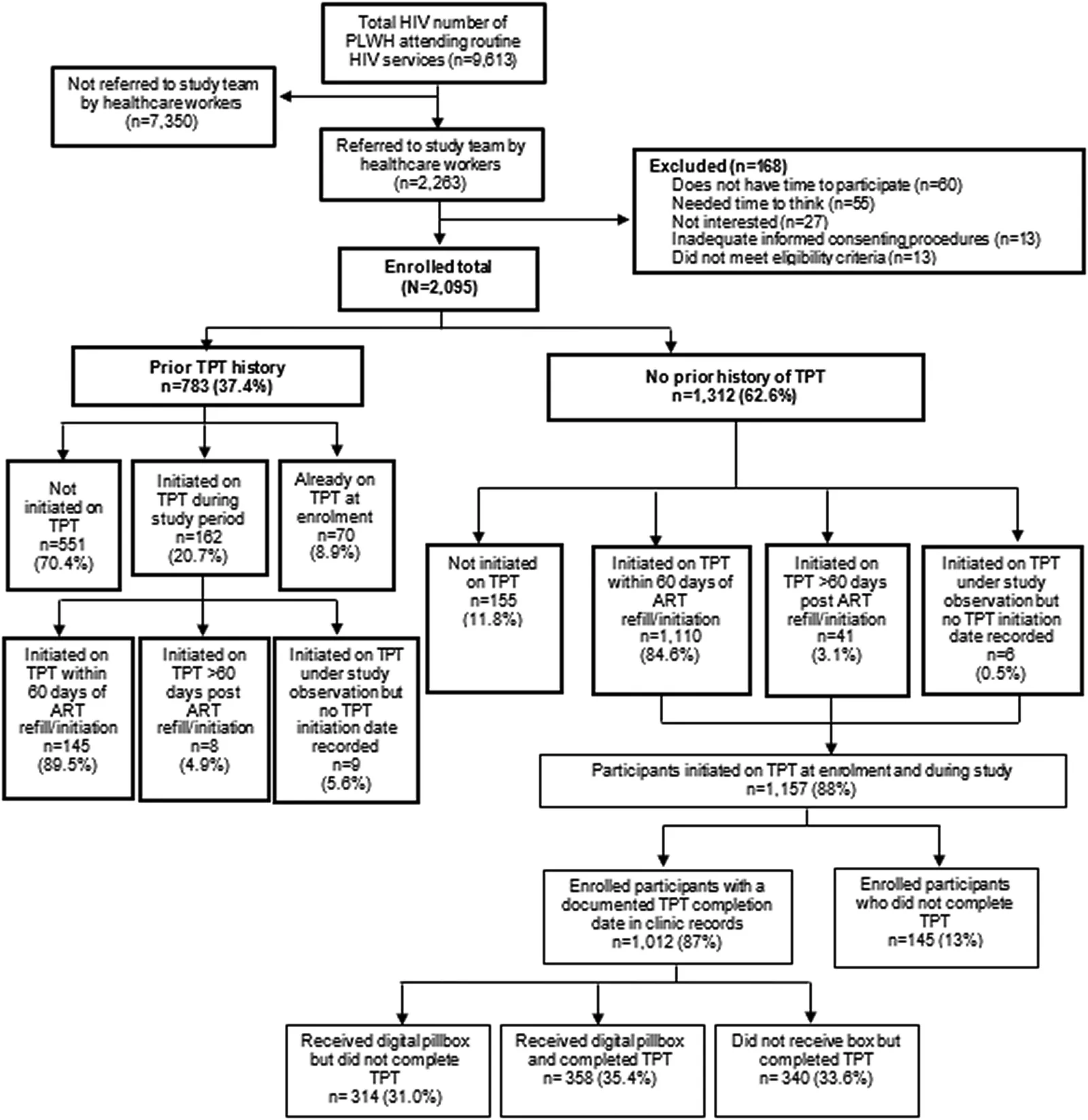
Flow chart for the optimizing tuberculosis-preventive treatment (TPT) delivery among PLWH (Opt4TPT) study. Abbreviations: ART, antiretroviral therapy; PLWH, people living with HIV.

Of the 2095 participants enrolled, most were women (n = 1489 [71.1%]), the median age was 42 years (IQR, 35–50 years), and about half reported food insecurity (n = 1098 [52.4%]) (Table 1). The majority were established on ART (n = 1828 [87.3%]); Among those, 284 (15.5%) reported receiving ART through community-based differentiated service delivery models. Nearly 14% of the participants (n = 287) reported a prior tuberculosis disease diagnosis (Table 1), with notable variation by country: 21.4% (n = 143) for Ethiopia, 7.2% (n = 53) for South Africa, and 13.1% (n = 91) for Zimbabwe (*P* < .001). TPT prior to enrollment was reported by 783 participants (37.4%); most with prior TPT were in Ethiopia (n = 535 [68.3%]).

**Table 1.** Baseline Demographic and Clinical Characteristics of Participants Living With Human Immunodeficiency Virus

Characteristic	Participants, No. (%)^a^		
	Total Cohort (N = 2095)	Received TPT^b^ (n = 1389)	No TPT^c^ (n = 706)
Sex			
Male	606 (28.9)	420 (69.3)	186 (30.7)
Female	1489 (71.1)	969 (65.0)	520 (35.0)
Age, median (IQR), y	42 (35–50)	42 (34–49)	42 (35–50)
Country			
Ethiopia	667 (31.8)	149 (22.3)	518 (77.7)
South Africa	733 (35.0)	562 (76.7)	171 (23.3)
Zimbabwe	695 (33.2)	678 (97.6)	17 (2.4)
Marital status			
Single	613 (29.3)	417 (68.0)	196 (32.0)
Married/cohabiting/not living with partner	886 (42.3)	624 (70.4)	262 (29.6)
Separated/divorced/widowed	596 (28.4)	348 (73.5)	248 (41.6)
Employment status			
Employed	521 (29.4)	315 (60.5)	206 (39.5)
Part time/informal/other	400 (19.1)	298 (74.5)	102 (25.5)
Self-employed	411 (19.6)	279 (67.9)	132 (32.1)
Unemployed	763 (36.4)	497 (65.1)	266 (34.9)
Food insecurity in past 4 wk			
Never/rarely	996 (47.5)	674 (67.7)	322 (32.3)
Sometimes/often	1097 (52.4)	714 (65.1)	383 (34.9)
Missing	2 (0.1)	1 (50.0)	1 (50.0)
Duration on ART at enrollment			
≤6 mo (Newly initiated)	267 (12.7)	248 (92.2)	19 (7.1)
>6 mo (Established)	1828 (87.3)	1141 (62.4)	687 (37.6)
Dolutegravir-based ART regimen at enrollment			
No	419 (20.0)	297 (70.9)	122 (29.1)
Yes	1676 (80.0)	1092 (65.2)	584 (34.8)
Self-reported tuberculosis history			
No	1808 (86.3)	1224 (67.7)	584 (32.3)
Yes	287 (13.1)	165 (57.5)	122 (42.5)
W4SS at study enrollment^d^			
No	1951 (93.2)	1307 (67.0)	644 (33.0)
Yes	144 (6.9)	82 (56.9)	62 (43.1)
Prior TPT history			
No	1312 (62.6)	1157 (88.2)	155 (11.8)
Yes	783 (37.4)	232 (29.6)	551 (70.4)
ART delivery through community-based DSD models^e^			
No	1794 (85.6)	1167 (65.1)	627 (34.9)
Yes	299 (14.3)	222 (74.2)	77 (25.8)
Missing	2 (0.1)	…	2 (100.0)
Viral load suppressed (<50 copies/mL)			
No	180 (8.6)	132 (73.3)	48 (26.7)
Yes	1458 (78.8)	932 (63.9)	526 (36.1)
Missing	457 (12.6)	325 (71.1)	132 (28.9)

Abbreviations: ART, antiretroviral therapy; DSD, differentiated service delivery; IQR, interquartile range; TPT, tuberculosis-preventive treatment; W4SS, World Health Organization–recommended 4-symptom screen.

^a^Data represent no. (%) of participants unless otherwise specified.

^b^Received TPT either before or during the study observation period.

^c^Data missing for 1 observation.

^d^Data on why TPT was not initiated and was not collected, as study staff relied on referral from healthcare workers. However, the most common reason for noninitiation was a recent history of TPT use.

^e^Among people living with human immunodeficiency virus established on ART (for >6 mo); DSD included community ART refill groups and family and community pick-up points).

### TPT Initiation

In total, 1389 of 2095 participants (63.0%) had received TPT either before or during the study period (Figure 1). Of the 2095 enrolled, 1312 had no prior documented TPT history and were considered eligible for TPT, of whom 88% (1157 of 1312) received TPT (Figure 2). The majority received TPT within 60 days of ART initiation or a refill visit (n = 1110 [84.6%]). Despite previous TPT history, 162 of 783 (20.7%) were reinitiated on TPT mostly within 60 days of ART refill (Figure 2). Most PLWH newly initiating ART were also initiated on TPT (248 of 267 [92.2%]) (Table 1).

**Figure 2.**
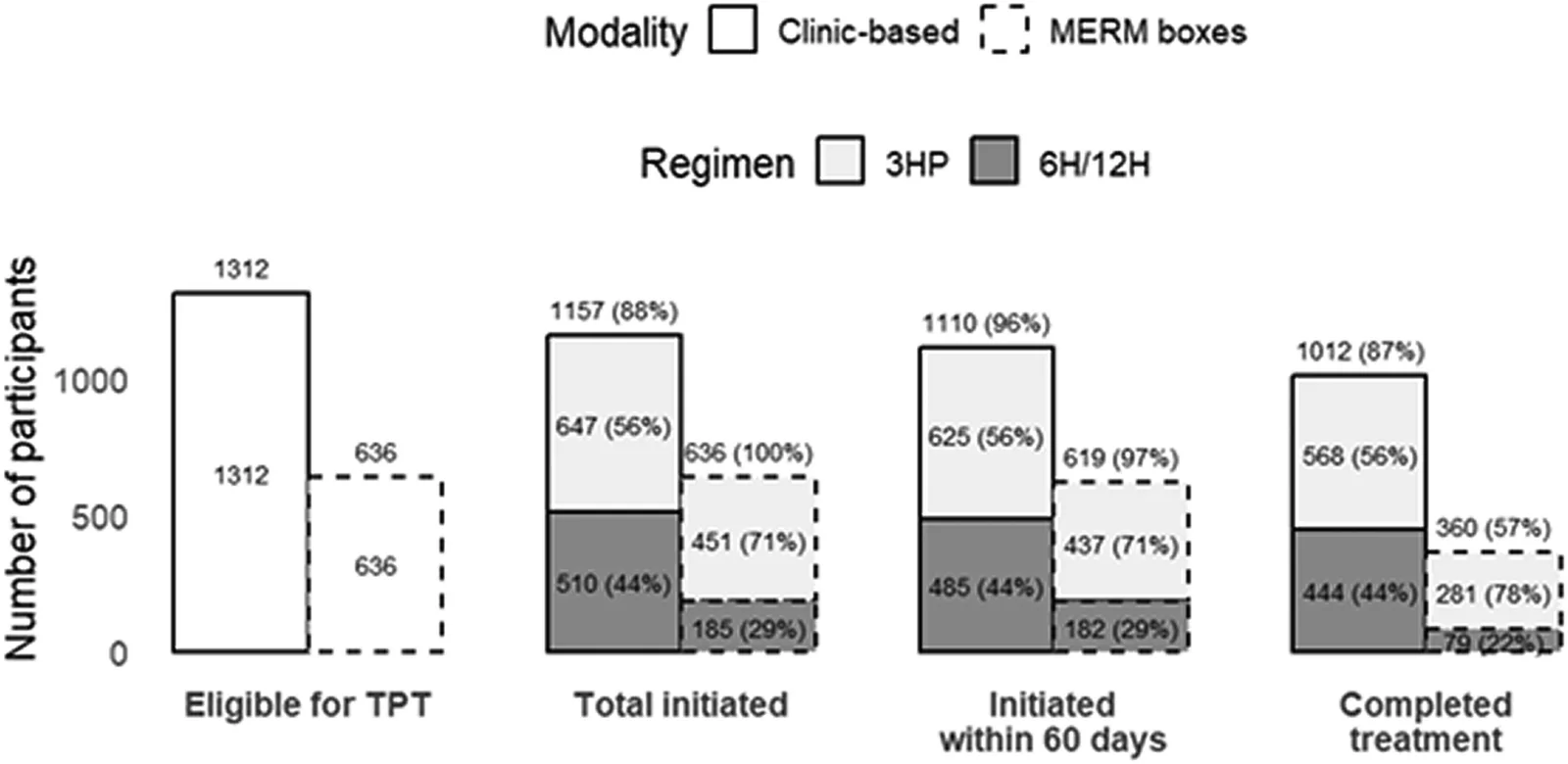
Tuberculosis-preventive treatment (TPT) cascade of care, overall, by modality and TPT regimen. Abbreviations: 3HP, rifapentine and isoniazid; 6H/12H, 6 or 12 months of isoniazid; MERM, medication event reminder monitor.

In total, 706 PLWH were not initiated on TPT, >70% (n = 551) due to a recently completed TPT regimen. In Ethiopia, for instance, most participants had completed TPT 1–3 years before enrollment (476 of 667 [71.3%]) and were considered not eligible for retreatment according to local guidelines. Among those without prior documented TPT history were 155 (11.8%) who did not receive TPT, and their reasons were undocumented.

Of the 1389 who received TPT, 716 (51.5%) were started on 3HP and 673 (48.5%) on 6H/12H. Most were initiated at study enrollment (1078 [77.6%]) and 67 (4.8%) within 7 days of enrollment. Another 162 (17.6%) were initiated >7 days after enrollment. while 70 (3.3%) were already on TPT at study entry.

### Factors Associated With TPT Initiation

TPT was more likely to be initiated among people without prior TPT use (adjusted incidence rate ratio [aIRR], 4.0 [95% CI, 2.08–7.69]; *P* < .001) and ≥1 tuberculosis symptom reported to study staff at enrollment (4.36; [2.37–8.02]; *P* = .001). Age, sex, and reporting food insecurity in the past month were not associated with TPT initiation (Supplementary File 1).

### TPT Completion Among Participants

Overall drop-off along the TPT cascade is illustrated in Figure 2 and in Supplementary File 2a, 2b, and 2c for country-specific summaries.

### Clinic-Based TPT Completion

TPT completion via clinic records showed that 1012 of 1157 participants (87.5%) (Figure 1) had a record of full dispensing of medications in the clinic files, meeting the clinic-based definition of TPT completion.

### MERM-Based Overall TPT Completion

Of the 880 participants who received TPT and were allocated an electronic pillbox, 733 were included in the MERM analysis. The participant characteristics of PLWH with a MERM box are summarized in Supplementary File 3. Exclusions were because devices never communicated with the server (n = 20); the participant was already on TPT when the device was allocated (n = 22); there were discrepancies in the TPT prescribing information between the digital pillbox data and the medical record abstractions (n = 23); the participant reported withdrawal, death, or transfer to another healthcare facility before TPT completion (n = 12); or the participant returned the box before TPT completion (n = 1). Overall, 406 (55.4%) had MERM evidence of having completed TPT (Figure 2), with completion rates of 42.1% among those who initiated 6H/12H (n = 101) and 61.9% among those who initiated 3HP (n = 305).

In adjusted Poisson analysis, participants who initiated 3HP were more likely to complete treatment than those prescribed 6H/12H (aIRR, 1.35 [95% CI, 1.25–1.54]), older participants (≥35 years) were more likely to complete treatment than younger participants (1.57 [1.25–1.96]), those reporting missing ≥1 TPT dose were less likely to complete TPT (0.77 [.64–.94])m and those with documented TPT completion in clinic records were approximately twice as likely to have completed TPT, as assessed by the digital pillbox (2.03 [1.44–2.85]) (Table 2). Duration on ART was not associated with TPT completion. Table 2 shows the associations with TPT completion based on MERM analysis of only those participants with TPT completion status. Sensitivity analysis results for all 880 PLWH who were allocated a MERM device are shown in Supplementary File 4.

**Table 2.** Factors Associated With Tuberculosis-Preventive Treatment Completion Among Participants Assigned a Digital Pillbox (n = 733)

Factor	Crude Analysis		Adjusted Analysis	
	IRR (95% CI)	*P* Value	aIRR (95% CI)	*P* Value
Age category				
<35 y	1 (Reference)	…	1 (Reference)	…
≥35 y	1.66 (1.26–2.19)	<.001	1.56 (1.24–1.95)	<.001
Sex				
Male	1 (Reference)	…	…	…
Female	0.92 (.75–1.13)	.41	…	…
Duration on ART				
Newly initiated	1 (Reference)	…	1 (Reference)	…
Established on ART	1.23 (.92–1.74)	.14	1.02 (.91–1.15)	.73
TPT regimen				
6H/12H	1 (Reference)	…	1 (Reference)	…
3HP	1.48 (1.18–1.85)	.001	1.36 (1.20–1.51)	<.001
Self-reported missed TPT doses				
No	1 (Reference)	…	1 (Reference)	…
Yes	0.77 (.61–.97)	.02	0.79 (.67–.95)	.01
Receiving ART from a community-based DSD model				
No	1 (Reference)	…	…	…
Yes	1.10 (.85–1.44)	.46	…	…
Reported adverse effects				
No	1 (Reference)	…	1 (Reference)	…
Yes	1.16 (.91–1.48)	.23	1.08 (1.05–1.25)	<.001
Reported food insecurity in past 4 wk				
No	1 (Reference)	…	…	…
Yes	1.05 (.87–1.28)	.61	…	…
Clinic-based TPT completion				
No	1 (Reference)	…	1 (Reference)	…
Yes	2.07 (1.32–3.24)	.002	2.03 (1.44–2.85)	<.001

Abbreviations: 3HP, rifapentine and isoniazid; 6H/12H, 6 or 12 months of isoniazid; aIRR, adjusted incidence rate ratio; ART, antiretroviral therapy; CI, confidence interval; DSD, differentiated service delivery; IRR, incidence rate ratio; TPT, tuberculosis-preventive treatment.

### Reported Symptoms During Follow-up by TPT Status

Overall, 360 of the 2095 (17.2%) participants reported a total of 576 symptoms to the study team: 198 (16.5%) from participants receiving 6H/12H, 141 (19.1%) from participants receiving 3HP, and 21 (13.5%) from those not on TPT at any time. Overall, the median time (IQR) since TPT initiation to reporting these symptoms was 8.5 (IQR, 1–30) days. Mild symptoms compatible with TPT adverse effects were more commonly reported with 3HP than with 6H/12H (190 [31.0%] vs 51 [11.0%], respectively; *P* < .001). Symptoms that led to missed TPT doses were reported in 25 of 360 participants (6.9%); separately, in a file review of symptoms recorded by clinicians, 146 participants reported a total of 161 symptoms. The most common symptoms documented by clinicians were symptoms suggestive of early hepatotoxicity among those receiving 3HP (n = 36 [3.8%]) and potential hypersensitivity reactions among those receiving 6H/12H (n = 36 [1.3%]) (Figure 3). No deaths were attributed to medication adverse effects. Figure 3 summarizes the symptoms reported to both study and clinic staff during follow-up.

**Figure 3.**
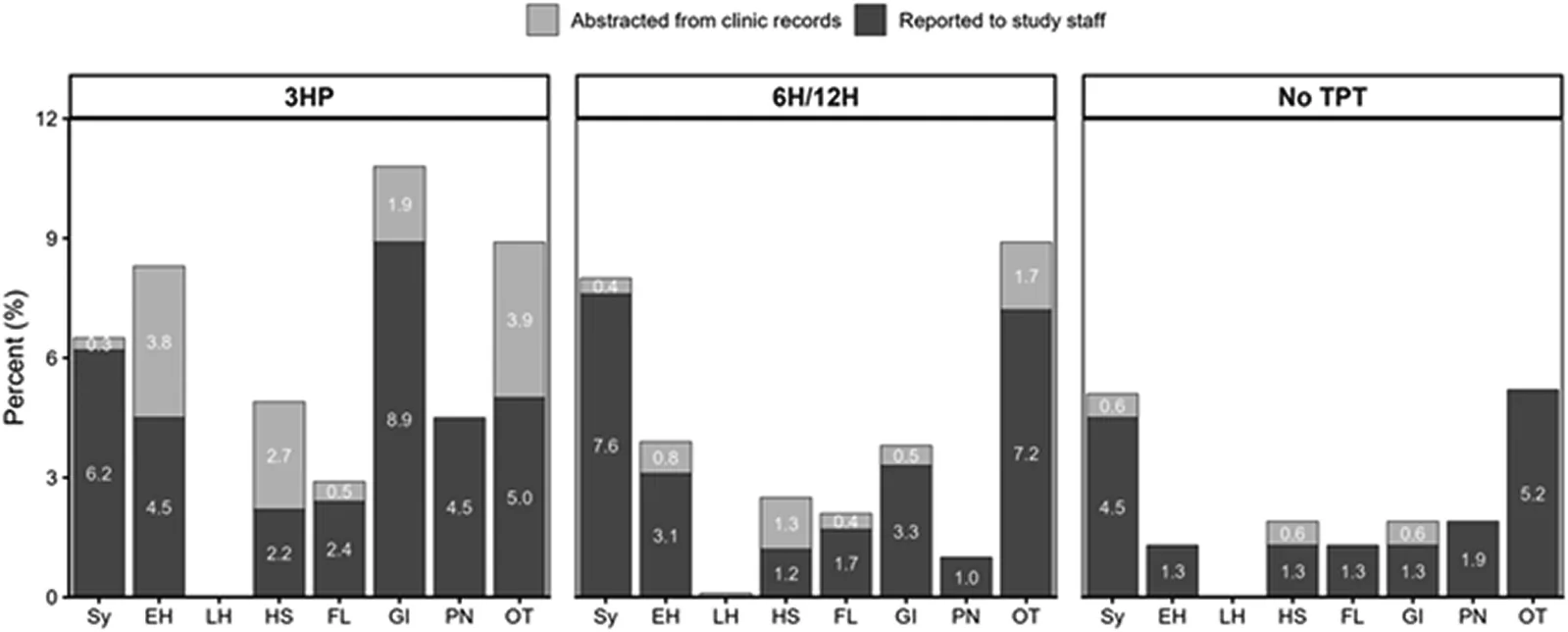
Frequency of symptoms reported to staff and clinically assessed, by tuberculosis-preventive treatment (TPT) regimen. Abbreviations: 3HP, rifapentine and isoniazid; 6H/12H, 6 or 12 months of isoniazid; EH, early hepatotoxicity; FL, flulike symptoms; GI, gastrointestinal symptoms; HS, hypersensitivities; LH, late hepatotoxicity; OT, other; PN, peripheral neuropathy; Sy, tuberculosis symptoms.

### Missed TPT Doses

Overall, 312 of 1157 participants receiving TPT (27%) reported missing ≥1 doses. The most common reasons were forgetting (n = 90 [36.0%]), time constraints (n = 21 [8.4%]), medication running out (n = 25 [10.0%]), and reported symptoms/adverse effects (n = 22 [8.8%]). More of the participants who reported missed doses due to adverse effects were receiving 3HP (15 of 22 [68.2%]) rather than 6H/12H (7 of 22 [31.8%]). Among those who completed TPT as assessed with the MERM box, 26.1% (204 of 782) were recorded to have missed ≥1 TPT dose.

### Tuberculosis Incidence Rate During Follow-up

During the 2 years of observation, 51 PLWH were diagnosed with tuberculosis. Four were diagnosed within 3 months of enrollment and were considered to have prevalent disease. Forty-seven were diagnosed 3–24 months after study enrollment during the 12- or 24-month microbiological testing or were routinely diagnosed by a healthcare worker at the study clinic. The incidence rate during this period was 1.27 (95% CI, .95–1.69) per 100 person years. Among these participants, 24 (51%) were asymptomatic with tuberculosis detected during sputum-based microbiological testing 12 or 24 months after enrollment. Although this finding was inconclusive, the crude tuberculosis incidence rate tended to be higher in PLWH established on ART (incidence rate ratio, 1.47 [95% CI, .53–4.10]) than those newly initiating ART. Tuberculosis disease incidence during the study period was lower among participants who completed TPT (aIRR, 0.39 [95% CI, .20–.78]; *P* = .01).

### Mortality Rates During Follow-up

Of 25 deaths recorded, 3 were related to tuberculosis and in participants who had no previous tuberculosis history and were started on 3HP during the study. Other causes of death included cancer (n = 6), complications of diabetes (n = 1), toxoplasmosis (n = 1), nonspecific infections (n = 4), natural causes (n = 1), a road accident (n = 1), and cardiac disease (n = 1); for 7 deaths no cause was documented. The all-cause mortality rate was higher in participants not on TPT than in those on any TPT (Supplementary File 5).

## DISCUSSION

We demonstrated that high levels of TPT initiation are feasible in routine settings: 80% of participants without prior TPT or recent tuberculosis treatment received TPT during the study period, 90% among those initiating ART. In addition, TPT was well tolerated and appeared to improve clinical outcomes, with a lower incidence rate of tuberculosis among those who were initiated on TPT and even lower rate among those who completed it. No deaths or severe morbid outcomes were attributable to TPT. A notable challenge was TPT completion as measured by MERM, with only roughly half of participants completing the regimen, with a higher proportion of those who received 3HP completing, compared with those receiving 6H/12H.

The proportion of participants who received TPT was higher than national reporting from each country [1] and a recent multiregional site survey in Africa, Asia and Latin America [20] but consistent with findings of several other studies [10, 12, 21–24]. Referral bias may have contributed to the overestimation of TPT initiation rates (80%–90%) compared with findings from other countries [25]. TPT initiation in the current study was associated with lack of TPT history, consistent with TPT delivery according to the respective national guidelines for each country. Contrary to guidance on starting TPT after ruling out active tuberculosis, our findings showed that TPT was initiated regardless of the presence of 1 of the WHO 4 symptoms. This discrepancy could be due to social desirability bias from PLWH reporting no symptoms to the clinician but reporting them to study staff preventing the ruling out of active tuberculosis before TPT initiation. The study population largely comprised PLWH established on ART. This is programmatically important, as HIV care in many settings is increasingly characterized by a growing number of long-term ART recipients. Therefore, these findings characterize the ability to provide TPT to this population, the ongoing high tuberculosis disease incidence rate, and the suggestion that TPT (especially completion) reduced tuberculosis disease incidence.

TPT including 3HP is well tolerated overall, with adverse effects infrequently leading to discontinuation, consistent with findings of clinical trials [17, 24, 26]. Interestingly, the positive association between reported adverse effects and treatment completion only became apparent after adjustment for other participant and programmatic factors. One possible explanation is that participants who reported adverse effects were more engaged with healthcare providers, creating opportunities for timely counseling, reassurance, and symptom management that encouraged them to remain on treatment. In contrast, participants who discontinued treatment early may have had limited contact with healthcare services and therefore fewer opportunities to report adverse events. This may have obscured the relationship in the unadjusted analysis, while adjustment for potential confounders revealed the independent association between reported adverse effects and treatment completion.

The higher frequency of mild self-limiting symptoms from 3HP compared with 6H/12H is consistent with reported rifapentine-based regimens. Only 14% of PLWH on 3HP missed doses, due to presumably mild adverse effects, reassuring clinicians that most symptoms can be managed without interrupting treatment. Adverse effects were reported by a larger proportion of participants on 3HP than on 6H/12H, a finding consistent with a systematic review of TPT-related adverse events [27]. A greater proportion of PLWH receiving 3HP reported symptoms to study staff than to clinic-based healthcare providers, suggesting that mild symptoms may be underrecognized during routine care. This may reflect limited opportunities for symptom assessment during clinic visits or greater willingness among participants to discuss minor symptoms with research staff. Although often self-limiting, failure to identify and manage mild symptoms may represent missed opportunities for counseling and early intervention, potentially affecting adherence and TPT completion.

Overall TPT completion in our study was high, which is consistent with findings from previous studies conducted among PLWH [28]. High completion rates were also reported in adult and child household contacts in India [29] and Eswatini [30], respectively. Such comparative findings should be cautiously interpreted due to differences in study populations, implementation settings, and mode of TPT completion assessment. Studies in routine programmatic settings, when operationalized, often meant that 3HP was documented as completed at the time of initial dispensing because the full regimen was often provided as a single package. For 6H/12H, 3-monthly dispensing was done. The high overall completion rate in routine settings is likely influenced by program definitions based on treatment completion rather than actual taking of medication. A study in South Africa, United States, Hong Kong, and Spain showed that isoniazid completion according to the standard of care was overestimated in South Africa and the United States [28], similar to our study’s finding.

Consistent with findings of previous studies, PLWH receiving 3HP were more likely to complete TPT than those receiving 6H/12H. The shorter treatment duration for 3HP likely reduces treatment fatigue and the cumulative burden of daily medication, making it easier for patients to complete the regimen. We sought to monitor TPT adherence using digital pillboxes to provide a patient-level view of adherence. This is in contrast to the routine clinical practice in the 3 study countries of reporting completion as having dispensed the full course of a regimen. Although the digital pillbox also has challenges, the overall completion rate was (55%), with higher completion rates observed for 3HP (63%) than for 6H (43%), which was plausible and consistent with prior studies [10, 17, 28, 31]. In addition, our findings show that individuals with documented TPT completion in clinic records were more likely to have completed treatment as assessed by the digital pillbox, suggesting reasonable concordance between routine documentation and objective adherence measures. However, incomplete concordance indicates that clinic records alone may overestimate true completion.

We also observed that being older and being on 3HP increased the likelihood of TPT completion. This is consistent with studies showing that shorter TPT regimens favor completion [10]. Despite reporting adverse effects, PLWH completed TPT, suggesting that most reported adverse effects were likely mild and manageable, allowing participants to continue treatment with appropriate clinical support. We also observed a lower rate of incident tuberculosis among those who completed TPT, suggesting a degree of accuracy for the adherence monitoring using the pillbox. Sensitivity analyses of all participants who received a digital pillbox produced findings similar to those with completion data only, suggesting that the observed determinants of TPT completion were robust. Our findings suggest that integrating objective adherence tools could strengthen monitoring and improve confidence in TPT program outcomes.

Nevertheless, the observed heterogeneity in TPT cascade performance across countries likely reflects differences in health system capacity (high healthcare staff turnover in Zimbabwe), regimen availability (lack of 3HP fixed dose combination doses in South Africa), implementation fidelity, and patient populations (with the majority of PLWH in Ethiopia excluded due to recent TPT history). Variations in service integration, healthcare worker practices, medication supply, and socioeconomic barriers may have contributed to differential losses across the cascade. These findings underscore the need for context-specific implementation strategies rather than a uniform approach to TPT delivery.

We observed a relatively high incidence of tuberculosis despite most of our participants being long-term ART recipients. This finding is consistent with prior reports of tuberculosis incidences between 0.58–4.2 per 100 person years among those on ART [32, 33]. Tuberculosis continues to be a burden in the long-term ART population. This reinforces the importance of routine tuberculosis testing among those on established ART. In addition, our finding of considerable asymptomatic tuberculosis were similar to those of prior studies globally [34–37]. Active case finding that is not guided by symptom screening only should be considered an important activity due to the high proportion of asymptomatic disease among people on long-term ART and a considerable proportion of PLWH initiated on TPT despite reporting at least one tuberculosis symptom.

Our study has important limitations. First, all participants were referred to study staff by healthcare workers, presenting a missed opportunity to objectively assess every potential participant for TPT eligibility. Study-defined TPT eligibility was estimated from the records and defined as having no record of TPT history before enrollment. Second, healthcare workers likely referred participants for screening into the study when they were prescribing TPT, leading to a biased sample with a high proportion initiating TPT. Notably, our participants represented only a quarter of all the PLWH receiving care from the study facilities. Referral bias may have resulted in a larger proportion of our study participants receiving TPT than the eligible clinic population. Notably, the demographics of our study population roughly reflect those of HIV cohorts in the study countries, suggesting that our participants likely represent the overall ART populations at the study clinics [38–41]. Implementation of the tuberculosis prevention study may have influenced routine practices within these clinics.

Third, the electronic monitoring box only recorded whether the device was opened and thus is not a direct measurement of medication ingestion. Some participants had >100% calculated adherence, which may reflect the use of the device for other storage needs. As such, our estimated completion levels may be an overestimate compared to completion in routine care. In addition, the electronic boxes were not large enough to fit a full course of most TPT regimens, so participants were expected to refill the device as needed. Thus, a participant could have achieved treatment completion without the device recording it they chose not to refill it. For such participants, our estimated adherence levels would underestimate completion. Finally, another limitation of our analysis is the potential for immortal time bias when comparing tuberculosis incidence between participants who completed TPT and those who did not. Because participants had to remain tuberculosis free until completing treatment to be classified as completers, the period between TPT initiation and completion was inherently allocated to the completer group. This may have led to an overestimation of the protective association between TPT completion and tuberculosis incidence. Future analyses using time-varying exposure methods would provide a more robust assessment of this relationship.

Our study also has certain strengths. First, we demonstrated real-world relevance by reflecting the actual conditions in routine care, highlighting the practical challenges and variations that are not observed in clinical trials. Second, the use of diverse settings in southern and eastern Africa with different background rates of tuberculosis improves the representativeness. Third, the prospective nature of the study allowed for the tracking of exposures and outcomes, supporting causal inference.

In conclusion, we observed high TPT initiation rate among people initiating ART and those on long-term ART, a high incidence rate of tuberculosis, suboptimal completion of TPT regimens, and an association between TPT completion and lower tuberculosis incidence. Further work is needed to assure high levels of TPT initiation and the development of practical approaches to assessing and supporting adherence to TPT within an integrated tuberculosis/HIV model. Given the high burden of asymptomatic tuberculosis and persistent tuberculosis in long-term ART users, tuberculosis prevention should be prioritized. Strengthening the delivery of TPT has potential to further reduce tuberculosis-associated morbidity and mortality rates among the full spectrum of PLWH, including those on long-term and effective ART.

## Supplementary Material

ofag595_Supplementary_Data

## Supplementary Data

Supplementary materials are available at *Open Forum Infectious Diseases* online. Consisting of data provided by the authors to benefit the reader, the posted materials are not copyedited and are the sole responsibility of the authors, so questions or comments should be addressed to the corresponding author.

## References

[1] World Health Organization . Global tuberculosis report 2024. Geneva: World Health Organization, 2024. https://www.who.int/teams/global-programme-on-tuberculosis-and-lung-health/tb-reports/global-tuberculosis-report-2024. Accessed 14 May 2025.

[2] Godfrey-Faussett P . Policy statement on preventive therapy against tuberculosis in people living with HIV. Geneva: World Health Organization, 1998.

[3] Danel C, Moh R, Gabillard D, et al; TEMPRANO ANRS 12136 Study Group. A trial of early antiretrovirals and isoniazid preventive therapy in Africa. N Engl J Med 2015; 373:808–22. 10.1056/NEJMoa1507198 26193126

[4] Uplekar M, Weil D, Lonnroth K, et al WHO's new end TB strategy. Lancet 2015; 385:1799–801. 10.1016/S0140-6736(15)60570-0 25814376

[5] Oxlade O, den Boon S, Menzies D, et al TB preventive treatment in high-and intermediate-incidence countries: research needs for scale-up. Int J Tuberc Lung Dis 2021; 25:823–31. 10.5588/ijtld.21.0293 PMC8504492 34615579

[6] World Health Organization . Guidelines on the management of tuberculosis in people living with HIV. Geneva: World Health Organization, 2021. https://www.who.int/publications/i/item/9789240031596. Accessed 14 May 2025.

[7] National Department of Health . National consolidated guidelines for the management of HIV in adults, adolescents, children and infants and prevention of mother-to-child-transmission. Pretoria: South African National Department of Health, 2023. https://knowledgehub.health.gov.za/system/files/elibdownloads/2023-04/National%2520Consolidated%2520Guidelines%252030062020%2520signed%2520PRINT%2520v7.pdf. Accessed 16 September 2025.

[8] Ministry of Health and Child Care . Guidelines for HIV prevention, testing and treatment of HIV in Zimbabwe. Harare, Zimbabwe: Ministry of Health and Child Care, 2022. https://www.prepwatch.org/wp-content/uploads/2023/08/Guidelines-for-HIV-Prevention-Testing-and-Treatment-of-HIV-in-Zimbabwe-August-2022.pdf. Accessed 16 September 2025.

[9] Federal Ministry of Health . National consolidated guidelines for comprehensive HIV prevention, care and treatment. Addis Ababa: Federal Ministry of Health, 2018. https://www.afro.who.int/sites/default/files/2019-04/National%20Comprehensive%20HIV%20Care%20%20Guideline%202018.pdf. Accessed 16 September 2025.

[10] Churchyard G, Cárdenas V, Chihota V, et al Annual tuberculosis preventive therapy for persons with HIV infection: a randomized trial. Ann Intern Med 2021; 174:1367–76. 10.7326/M20-7577 34424730

[11] Martinson NA, Barnes GL, Moulton LH, et al New regimens to prevent tuberculosis in adults with HIV infection. N Engl J Med 2011; 365:11–20. 10.1056/NEJMoa1005136 PMC3407678 21732833

[12] Semitala FC, Kadota JL, Musinguzi A, et al Completion of isoniazid-rifapentine (3HP) for tuberculosis prevention among people living with HIV: interim analysis of a hybrid type 3 effectiveness-implementation randomized trial. PLoS Med 2021; 18:e1003875. 10.1371/journal.pmed.1003875 PMC8726462 34914696

[13] World Health Organization . WHO Operational handbook on tuberculosis. Module 1: prevention—tuberculosis preventive treatment. Geneva: World Health Organization, 2020.

[14] World Health Organization . Overview of WHO recommendations on HIV and sexually transmitted infection testing, prevention, treatment, care and service delivery. Geneva: World Health Organization, 2025. https://iris.who.int/bitstream/handle/10665/381896/B09471-eng.pdf?sequence=1. Accessed 15 September 2025.

[15] Churchyard GJ, Cardenas V, Chihota V, et al, eds. Effectiveness of 3HP annually vs once for HIV-positive people: the WHIP3TB trial. Presented at: Conference on Retrovirus and Opportunistic Infections (CROI); 8–11 March 2020; Virtual.

[16] Sterling TR, Scott NA, Miro JM, et al Three months of weekly rifapentine and isoniazid for treatment of *Mycobacterium tuberculosis* infection in HIV-coinfected persons. AIDS 2016; 30:1607–15. 10.1097/QAD.0000000000001098 PMC4899978 27243774

[17] Sterling TR, Villarino ME, Borisov AS, et al Three months of rifapentine and isoniazid for latent tuberculosis infection. N Engl J Med 2011; 365:2155–66. 10.1056/NEJMoa1104875 22150035

[18] Swindells S, Ramchandani R, Gupta A, et al One month of rifapentine plus isoniazid to prevent HIV-related tuberculosis. N Engl J Med 2019; 380:1001–11. 10.1056/NEJMoa1806808 PMC6563914 30865794

[19] StataCorp . Stata Statistical Software. Release 18. College Station, TX: StataCorp, 2019.

[20] Schmutz R, Banholzer N, Shearer K, et al The implementation of tuberculosis preventive therapy in HIV care clinics in Africa, Asia and Latin America: a multiregional site survey. BMJ Glob Health 2026; 11:e018469. 10.1136/bmjgh-2024-018469 PMC12853508 41592909

[21] Mapingure MP, Zech JM, Hirsch-Moverman Y, et al Integrating 3HP-based tuberculosis preventive treatment into Zimbabwe's Fast Track HIV treatment model: experiences from a pilot study. J Int AIDS Soc 2023; 26:e26105. 10.1002/jia2.26105 PMC10281638 37339341

[22] Roscoe C, Lockhart C, de Klerk M, et al Evaluation of the uptake of tuberculosis preventative therapy for people living with HIV in Namibia: a multiple methods analysis. BMC Public Health 2020; 20:1838. 10.1186/s12889-020-09902-z PMC7708912 33261569

[23] Semitala FC, Kadota JL, Musinguzi A, et al Comparison of 3 optimized delivery strategies for completion of isoniazid-rifapentine (3HP) for tuberculosis prevention among people living with HIV in Uganda: a single-center randomized trial. PLoS Med 2024; 21:e1004356. 10.1371/journal.pmed.1004356 PMC10914279 38377166

[24] Winters N, Belknap R, Benedetti A, et al Completion, safety, and efficacy of tuberculosis preventive treatment regimens containing rifampicin or rifapentine: an individual patient data network meta-analysis. Lancet Respir Med 2023; 11:782–90. 10.1016/S2213-2600(23)00096-6 PMC11068309 36966788

[25] Bastos ML, Melnychuk L, Campbell JR, Oxlade O, Menzies D. The latent tuberculosis cascade-of-care among people living with HIV: a systematic review and meta-analysis. PLoS Med 2021; 18:e1003703. 10.1371/journal.pmed.1003703 PMC8439450 34492003

[26] Menzies D, Adjobimey M, Ruslami R, et al Four months of rifampin or nine months of isoniazid for latent tuberculosis in adults. N Engl J Med 2018; 379:440–53. 10.1056/NEJMoa1714283 30067931

[27] Melnychuk L, Perlman-Arrow S, Lisboa Bastos M, Menzies D. A systematic review and meta-analysis of tuberculous preventative therapy adverse events. Clin Infect Dis 2023; 77:287–94. 10.1093/cid/ciad246 37125482

[28] Scott NA, Sadowski C, Vernon A, et al Using a medication event monitoring system to evaluate self-report and pill count for determining treatment completion with self-administered, once-weekly isoniazid and rifapentine. Contemp Clin Trials 2023; 129:107173. 10.1016/j.cct.2023.107173 PMC11078335 37004811

[29] Sharma N, Bakshi R, Basu S, Zode M, Arora R, Khanna A. Implementation of tuberculosis preventive therapy with INH-rifapentine (3HP) for latent tuberculosis infection management in household tuberculosis contacts in India: a prospective study. Trop Med Int Health 2023; 28:890–900. 10.1111/tmi.13940 37864386

[30] Sandoval M, Mtetwa G, Devezin T, et al Community-based tuberculosis contact management: caregiver experience and factors promoting adherence to preventive therapy. PLoS Glob Public Health 2023; 3:e0001920. 10.1371/journal.pgph.0001920 PMC10348572 37450473

[31] Alsdurf H, Hill PC, Matteelli A, Getahun H, Menzies D. The cascade of care in diagnosis and treatment of latent tuberculosis infection: a systematic review and meta-analysis. Lancet Infect Dis 2016; 16:1269–78. 10.1016/S1473-3099(16)30216-X 27522233

[32] Sendagire I, Ssempijja V, Ndyanabo A, et al Incidence and risk factors for tuberculosis at a rural HIV clinic in Uganda, 2012–2019: a retrospective cohort study. BMC Public Health 2025; 25:1882. 10.1186/s12889-025-23090-8 PMC12096586 40405106

[33] Suthar AB, Lawn SD, del Amo J, et al Antiretroviral therapy for prevention of tuberculosis in adults with HIV: a systematic review and meta-analysis. PLoS Med 2012; 9:e1001270. 10.1371/journal.pmed.1001270 PMC3404110 22911011

[34] Naidoo K, Moodley MC, Hassan-Moosa R, et al Recurrent subclinical tuberculosis among antiretroviral therapy-accessing participants: incidence, clinical course, and outcomes. Clin Infect Dis 2022; 75:1628–36. 10.1093/cid/ciac185 PMC9617574 35247054

[35] Bajema KL, Bassett IV, Coleman SM, et al Subclinical tuberculosis among adults with HIV: clinical features and outcomes in a South African cohort. BMC Infect Dis 2019; 19:14. 10.1186/s12879-018-3614-7 PMC6321698 30611192

[36] Frascella B, Richards AS, Sossen B, et al Subclinical tuberculosis disease—a review and analysis of prevalence surveys to inform definitions, burden, associations, and screening methodology. Clin Infect Dis 2020; 73:e830–41. 10.1093/cid/ciaa1402 PMC8326537 32936877

[37] Teo AKJ, MacLean EL-H, Fox GJ. Subclinical tuberculosis: a meta-analysis of prevalence and scoping review of definitions, prevalence and clinical characteristics. Eur Respir Rev 2024; 33:230208. 10.1183/16000617.0208-2023 PMC11078153 38719737

[38] Joint United Nations Programme on HIV/AIDS . The urgency of now: AIDS at a crossroads. Geneva: UNAIDS, 2024. https://www.unaids.org/sites/default/files/media_asset/2024-unaids-global-aids-update_en.pdf. Accessed 9 October 2025.

[39] Pulerwitz J, Gottert A, Kahn K, et al Gender norms and HIV testing/treatment uptake: evidence from a large population-based sample in South Africa. AIDS Behav 2019; 23:162–71. 10.1007/s10461-019-02603-8 PMC6773668 31359218

[40] Seidu AA, Oduro JK, Ahinkorah BO, et al Women's healthcare decision-making capacity and HIV testing in sub-Saharan Africa: a multi-country analysis of demographic and health surveys. BMC Public Health 2020; 20:1592. 10.1186/s12889-020-09660-y PMC7583279 33092556

[41] Staveteig S, Wang S, Head SK, Bradley SEK, Nybro E. Demographic patterns of HIV testing uptake in sub-Saharan Africa. Calverton, MD: ICF International, 2013.

